# Manipulation of Emergent Collective Excitations via Composition Control in Mixed MPX_3_ Correlated 2D Antiferromagnets

**DOI:** 10.1002/advs.202517378

**Published:** 2025-12-12

**Authors:** Cong Tai Trinh, Na Liu, Rabindra Basnet, Dinesh Upreti, Rijan Karkee, Vigneshwaran Chandrasekaran, Andrew C. Jones, Michael T. Pettes, Thuc T. Mai, Michael A. Susner, Jin Hu, Rahul Rao, Han Htoon

**Affiliations:** ^1^ Center for Integrated Nanotechnologies Materials Physics and Applications Division Los Alamos National Laboratory Los Alamos NM 87545 USA; ^2^ Department of Physics University of Arkansas Fayetteville AR 72701 USA; ^3^ Department of Physics Morgan State University Baltimore MD 21251 USA; ^4^ Materials and Manufacturing Directorate Air Force Research Laboratory Wright‐Patterson Air Force Base OH 45433 USA; ^5^ BlueHalo Inc. Dayton OH 45432 USA; ^6^ Institute for Nanoscience and Engineering MonArk NSF Quantum Foundry and Smart Ferroic Materials Center University of Arkansas Fayetteville AR 72701 USA; ^7^ Present address: Department of Chemistry Purdue University West Lafayette IN 47907 USA; ^8^ Present address: Quantum Group Materials Physics and Applications Division Los Alamos National Laboratory Los Alamos NM 87545 USA

**Keywords:** 2D magnet, charge transfer, Fano resonance, magnetic exciton, magnon, NiPS_3,_ photoluminescence, raman spectroscopy

## Abstract

Transition metal (i.e., Mn, Fe, Cr) and chalcogen (Se) substituents are introduced into single‐crystalline NiPS_3_, and the evolution of the two emergent quasi‐particle excitations characteristic to the XXZ correlated antiferromagnetism of NiPS_3_ (i.e., spin orbit entangled exciton (SOX) and two‐magnon scattering (2M )) are investigated as functions of substituent concentration through comprehensive room‐ and low‐temperature photoluminescence (PL) and Raman spectroscopy studies. These findings are further correlated with the magnetic properties of the same set of compounds reported in prior studies. The work revealed that the SOX emission intensities and linewidths are mainly controlled by the magnetic anisotropy and spin orientations, and are strongly suppressed by the introduction of substituents. The suppression depends on the type of substituent, with Fe affecting the SOX emission more than Mn and Cr. The 2 m scattering is linked to short‐range correlations and exhibits greater resiliency against metal atom substitution. While the 2M  peak at low temperature gets suppressed and red‐shifted in frequency with increasing concentrations of all the substituents, Fe induces the weakest suppression compared to all other substituents. Altogether, these findings revealed the introduction of substituents as a powerful route to control the emergent collective excitations in NiPS_3_ and mixed‐MPX_3_ materials.

## Graphical abstract

Transition metal and chalcogen substituents are introduced into 2D antiferromagnet, NiPS_3_, and comprehensive photoluminescence and Raman spectroscopy studies are conducted to investigate how spin orbit entangled exciton and two magnon scattering evolve as functions of substituent concentration. Findings revealed the introduction of substituents as a powerful route to control the emergent collective excitations.

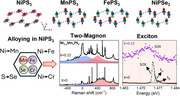

## 1 Introduction

Ternary metal phosphorous chalcogenides with the general formula MPX_3_, where M═Ni, Fe, Co, Mn, V, Cr, and X═S, Se, respectively, have recently been ushered to the forefront of 2D materials research.^[^ ^1^, ^2^, ^3^ ^]^ The surge of interest in this material system originates from the fact that they belong to a rare class of 2D correlated antiferromagnets where all three different antiferromagnetic (AFM) orders −Ising, Heisenberg, and XXZ− can be realized with a judicious choice of transition metal and chalcogen ions.^[^ ^1^, ^2^, ^3^ ^]^ Recent studies have shown that a complex interplay of charge, spin, orbital, and lattice degrees of freedom uniquely allowed by the MPX_3_ structure has led to the emergence of fascinating many‐body phenomena as well as opto‐spintronic and quantum information processing functionalities. These breakthroughs span from discoveries of pressure‐induced superconductivity,^[^ ^4^ ^]^ coherent spin‐orbit entangled excitons,^[^ ^5^, ^6^, ^7^ ^]^ hybrid exciton‐polaritons,^[^ ^8^ ^]^ exciton‐phonon,^[^ ^5^ ^]^ spin‐phonon,^[^ ^9^ ^]^ and exciton‐magnon bound states^[^ ^10^, ^11^ ^]^ to demonstrations of proximity‐induced chiral quantum light emission,^[^ ^12^ ^]^ ultra‐fast control of magnetic anisotropy^[^ ^13^, ^14^ ^]^ and a transient metallic state that preserves long‐range antiferromagnetism.^[^ ^15^ ^]^

Among these discoveries, one that stands out is the emergence of coherent spin‐orbit‐entangled exciton (SOX) emission in NiPS_3_ at 1.47 eV, with an ultra‐narrow linewidth (<0.4 meV) at temperatures below 50K, well below the Néel temperature (T_N_ ≈155 K).^[^ ^5^, ^6^, ^7^ ^]^ This emission is attributed to a spin‐or bit‐entangled exciton state that arises intrinsically from many‐body states of the Zhang–Rice singlet formed by the correlation between a localized *d* orbital of the Ni^2+^ ions and the *p* orbitals of the neighboring S ligands.^[^ ^6^ ^]^ With the assistance of AFM order, this exciton reaches a coherent state and gives rise to the atomically sharp emission lines. While this assignment is widely accepted and supported by recent magneto‐optical studies,^[^ ^16^, ^17^ ^]^ other experimental as well as theoretical studies^[^ ^18^, ^19^, ^20^, ^21^ ^]^ have produced evidence suggesting the origin of the emission to be either the intrinsic band structure^[^ ^22^ ^]^ or defect‐bound states in NiPS_3_.^[^ ^23^ ^]^ Earlier studies have further shown that this excitonic transition exhibits a strong degree of linear polarization that reflects the zigzag AFM order of NiPS_3_.^[^ ^5^, ^6^, ^7^ ^]^ Controversy again arose when later studies assigned the spin orientation as a factor determining the direction of the linear polarization.^[^ ^23^ ^]^ These controversies demand further investigation of the SOX emission.

**Figure 1:**
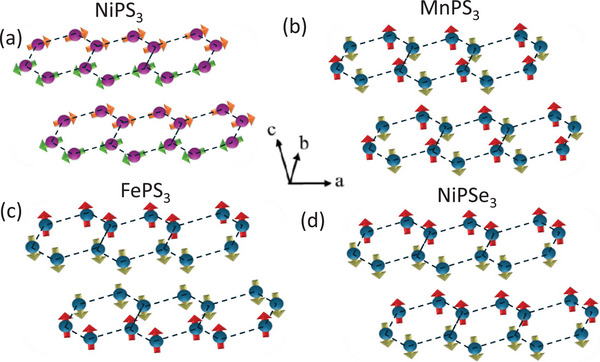
Magnetic structures of NiPS_3_ a), MnPS_3_ b), FePS_3_ c), and NiPSe_3_ d). Only the magnetic transition metal layer is shown.

Since the key characteristics of this SOX emission, namely emission linewidth and degree and orientation of linear polarization are strongly correlated with the spin orientation and/or zigzag AFM order of NiPS_3_, it would be particularly interesting to investigate how these characteristics would evolve when the spin orientation and AFM order are changed through the introduction of metal and chalcogen substituents. Mixed MPX_3_ compounds such as Ni_1‐x_Mn_x_PS_3_, Ni_1‐x_Fe_x_PS_3_, and NiPS_3‐x_Se_x_ offer a unique opportunity to address this issue. As illustrated in **Figure** **1**, the spin order in each AFM state for NiPS_3_, MnPS_3_, FePS_3_ and NiPSe_3_ is different, resulting in different AFM orders. In NiPS_3_ the spins are ordered in a zigzag structure with nearly in‐plane easy axis resulting in an XXZ‐type antiferromagnet. While FePS_3_ shares the zigzag‐type magnetic structure, out‐of‐plane alignment of spins leads to Ising‐type antiferromagnetism. MnPS_3_ is a Heisenberg antiferromagnet with spins aligned out‐of‐plane and slightly canted but arranged in Néel‐type ordering. While the magnetic structure of NiPSe_3_ is not well understood due to the difficulty in its synthesis, density functional theory calculations suggest a zigzag‐type AFM structure with out‐of‐plane spin orientations.^[^ ^24^, ^25^ ^]^ Similarly, while the isovalent substitution of Ni with Cr is also difficult and the magnetic structure of CrPS_3_ is unknown, a recent work has demonstrated that Cr substitution (up to 9%) effectively alter the magnetic isotropy while preserving the host lattice structure.^[^ ^26^ ^]^ Therefore, a tuning of composition (i.e., *x*) in Ni_1‐x_Mn_x_PS_3_, Ni_1‐x_Fe_x_PS_3_, Ni_1‐x_Cr_x_PS_3,_ and NiPS_3‐x_Se_x_ will enable control over the spin orientation and type of antiferromagnetism. While the synthesis and studies of magnetic properties of these mixed MPX_3_ have been reported,^[^ ^24^, ^26^, ^27^, ^28^, ^29^, ^30^, ^31^ ^]^ the evolution of SOX has only been investigated in the Ni_1‐x_Cd_x_PS_3_ and Ni_1‐x_Mn_x_S_3_ compounds, revealing strong quenching of SOX in both cases.^[^ ^32^, ^33^ ^]^ A study comparing the evolution of SOX with substituent concentration in different mixed MPX_3_ compounds and correlating the findings with the evolution of magnetic properties reported in earlier studies are still essential for a comprehensive understanding.

In a parallel development, excitation of spin and lattice degree of freedoms and their hybridized quasiparticle states in MPX_3_ compounds have been extensively investigated with Raman spectroscopy. Both the first‐order magnon (collective excitation of electron spins in a magnetically ordered state) and the second‐order two‐magnon excitations scatter light and are observable in the Raman scattering process. Additionally, phonon modes may exhibit asymmetric lineshapes due to coupling between the vibrations and magnons or the electronic continua of the charged metal ions in the MPX_3_ compounds. While the first order magnon is only observable in the AFM state, the two‐magnon (2M ) excitation survives well above *T*_N_ and can also be observed as a broad continuum in the room temperature Raman spectrum.^[^ ^34^, ^35^, ^36^, ^37^ ^]^ This peak appears ≈550 cm^−1^ in NiPS_3_ and couples strongly to the high‐frequency *E*_g_ symmetry phonon mode ≈560 cm^−1^.^[^ ^38^, ^39^ ^]^ At room temperature, this mode displays an asymmetric lineshape due to Fano resonance (in this case due to the coupling between this mode and the two‐magnon continuum), which becomes more pronounced below *T*_N_.^[^ ^38^ ^]^ Recently, low‐energy Raman modes that can be attributed to one magnon exciation are also observed.^[^ ^40^ ^]^

In addition to the high‐frequency Raman mode, two other Raman modes exhibit the asymmetric Fano lineshape in NiPS_3_. The first is an intense mode (∼255 cm^−1^) involving out‐of‐plane vibrations of S atoms. This mode exhibits excitation energy‐dependent resonance behavior and was recently found to couple strongly to the AFM order in NiPS_3_.^[^ ^9^ ^]^ In fact, this mode is common to all the MPS_3_ compounds but exhibits the highest asymmetry (Fano parameter) in NiPS_3_. This was attributed to the shortest distance (among the MPS_3_ compounds) between the Ni^2+^ and S atoms in the NiPS_3_ structure. As a result, NiPS_3_ develops a negative charge transfer state due to electron transfer from S to Ni^2+^ and manifests as a second continuum peak at low frequencies in its Raman spectrum due to the charge transfer.^[^ ^41^ ^]^ The last phonon mode that exhibits Fano asymmetry in NiPS_3_ couples to this low‐frequency charge transfer continuum. The mode, ∼175 cm^−1^, corresponds to in‐plane vibrations of S and P atoms. Interestingly, of the three asymmetric Raman modes at room temperature, the 175 and 255 cm^−1^ modes become more symmetric below *T*_N_ along with the disappearance of the low‐frequency charge transfer continuum (CTC).^[^ ^9^, ^41^ ^]^ On the other hand, the high‐frequency mode ≈560 cm^−1^ becomes more asymmetric, with a concomitant increase in the two‐magnon continuum intensity.^[^ ^38^ ^]^

The continua and Fano resonances in NiPS_3_ are affected by site doping in a number of significant ways. Prior studies on Fe‐doped NiPS_3_ (Ni_1‐x_Fe_x_PS_3_) have reported that the two magnon continuum red‐shifts in frequency and decreases in intensity with increasing Fe content.^[^ ^42^ ^]^ Surprisingly, these effects can be seen deep into the Fe‐rich phase (up to x = 0.8) while the XXZ‐magnetic order is supressed at much lower Fe fractions (x = 0.1). The Raman studies indicate that the short‐range spin correlations decrease slowly with increasing Fe^2+^ content, while the static magnetism is quickly switched from XXZ to an Ising‐like behavior. Doping of NiPS_3_ with non‐magnetic Zn showed the diminishing of the charge transfer continuum with temperature and a concomitant increase in the asymmetry of the 176 cm^−1^ mode.^[^ ^40^ ^]^ This was attributed to rebalancing of the charge transfer between the S atoms and Ni^2+^ ions due to the inclusion of the Zn^2+^ ions with their fully occupied *d* orbitals.

Taken together, these studies reveal that Raman spectroscopy is a powerful tool to investigate magnons, phonons and their hybridized bound states, which evolve with the compositional tuning of magnetic anisotropy and correlations in mixed MPX_3_ compounds. A recent study showed that the SOX emission in NiPS_3_ is also bound strongly to the out‐of‐plane A_1g_ phonon mode ∼255 cm^−1^.^[^ ^5^ ^]^ Thus, the sensitivity of the phonon modes to the short‐range correlations can be exploited to shine new light on the SOX in NiPS_3_ by systematically analyzing its evolution as a function of chemical composition in correlation with that of the 2 m and Fano resonances revealed by the Raman studies. To the best of our knowledge, such analyses across different mixed MPX_3_ compounds have not been reported.

Aiming to fill these gaps, we conducted temperature‐dependent photoluminescence(PL) and Raman spectroscopy studies on a set of mixed MPX_3_ compounds: Ni_1‐x_Mn_x_PS_3_, Ni_1‐x_Fe_x_PS_3_, Ni_1‐x_Cr_x_PS_3_ and NiPS_3‐x_Se_x_ with a wide range of compositions. A direct comparison among substitution‐dependent evolutions of the SOX (PL) and 2M  (Raman) peaks at room‐ and low‐temperature revealed multiple intriguing behaviors that contrast from one type of substituent to another. By correlating these behaviors with the results of prior studies on magnetic properties of the mixed MPX_3_ compounds, we can explain these behaviors as the consequence of the influence of substituents on magnetic anisotropy of the long‐range static order and short‐range correlations that separately control SOX emission and 2M scattering, respectively.

## 2 Results

### 2.1 Alloying NiPS_3_ Compositions and Correlation with Reported Magnetic Properties

All the MP*X*_3_ compounds used in this work were synthesized using chemical vapor transport (CVT), which has been proven to be a highly efficient method for synthesizing these materials and their metal‐ and chalcogen‐substituted compounds.^[^ ^27^ ^]^ The structure and compositions of the obtained crystals are characterized by X‐ray diffraction (XRD) (Figure S7, Supporting Information) and energy dispersive x‐ray spectroscopy (EDS) (Table S1, Supporting Information), respectively. The details of sample growths and characterizations have been summarized in the Experimental Section. For isovalent Mn and Fe substitution for Ni, the CVT method successfully produces single crystals covering the entire Ni_1‐x_Mn_x_PS_3_^[^ ^28^, ^29^, ^31^ ^]^ and Ni_1‐x_Fe_x_PS_3_j^[^ ^30^ ^]^ composition range 0 ≤ x ≤ 1. For Cr substitution for Ni, however, the isovalent substitution is found to be difficult and a maximum Cr content of 9% is achievable.^[^ ^26^ ^]^ Further Cr addition leads to phase separation and the formation of CrPS_4_, another compound with crystal structure and bonding fundamentally different from MPX_3_ family and therefore not relevant to investigate in this study. For chalcogen S‐Se substitution, similarly, owing to the difficulty in producing NiPSe_3_ single crystals, the CVT synthesis yielded good single crystals up to only x = 1.3 in NiPS_3‐x_Se_x_.^[^ ^24^ ^]^ In this work, we performed optical studies based on the availability of sample compositions for both metal‐ and chalcogen‐substituted NiPS_3_. We studied samples in the whole composition range from x = 0 to 1 for Ni_1‐x_Mn_x_PS_3_ and Ni_1‐x_Fe_x_PS_3_, and limited compositions ranging from x = 0 to 0.09 and x = 0 to 0.2 have been measured for Ni_1‐x_Cr_x_PS_3_ and NiPS_3‐x_Se_x_, respectively. Such a wide range of metal‐ and chalcogen‐substituted alloys can be attributed to the isostructural nature of MPX_3_ materials, which allows the synthesis of polymetallic and polychalcogenide MPX_3_ compounds. The MPX_3_ materials exhibit common structural characteristics in which metal atoms M are arranged in a honeycomb lattice and sandwiched by P and X atoms, where P forms P‐P dimers perpendicular to the hexagonal metal plane and bonded with three *X* to form (P_2_ *X*_6_)^4−^ bipyramids filling the center of the M honeycomb lattice. Our optical studies were performed on thick (bulk‐like) exfoliated flakes that ensured a clean cleaved surface providing the highest signal‐to‐noise.

The composition tunability of MPX_3_ provides a perfect platform for systematic engineering of magnetic properties depending on the composition stoichiometry. Both metal and chalcogen substitutions have been established as powerful techniques to modulate magnetism in MPX_3_. Because MnPS_3_^[^ ^43^ ^]^ and FePS_3_^[^ ^44^ ^]^ possess distinct AFM structures as compared to NiPS_3_,^[^ ^45^ ^]^ substitutions of Mn and Fe for Ni can lead to effective tuning of magnetic properties although the magnetic ground state remains AFM.^[^ ^28^, ^29^, ^30^ ^]^ In Ni_1‐x_Mn_x_PS_3_, substituting Mn for Ni is found to rotate the magnetic easy axis from the nearly in‐plane direction for NiPS_3_ to the out‐of‐plane direction for MnPS_3_,^[^ ^24^, ^29^ ^]^ as manifested by the drastic modification of the spin‐flop (SF) transition with only a few precent of substitutions.^[^ ^24^ ^]^ Because an SF transition occurs when magnetic field components along the easy axis exceed a certain critical field, it can be used as a convenient tool to examine the evolution of magnetic anisotropy and magnetic structure with substitution. NiPS_3_ displays an SF transition under an in‐plane field (*H*//*ab*) of ≈6 T at 2 K, which is consistent with its almost in‐plane moment orientation as shown in Figure 1a. On the other hand, in MnPS_3_, a SF transition occurs under an out‐of‐plane magnetic field (*H*⊥*ab*) of ≈3.5 T at 2 K, in line with its magnetic structure depicted in Figure 1b. A light Ni‐Mn substitution of a few percent substantially affects the SF transition for both end compounds MnPS_3_ and NiPS_3_.^[^ ^28^ ^]^ For Fe‐substituted NiPS_3_, a transition from XXZ‐ to Ising‐type anisotropy is seen with only 10% Fe substitution in NiPS_3_,^[^ ^30^ ^]^ which might be attributed to highly anisotropic Ising‐type magnetism for FePS_3_. Furthermore, the Cr substitution is found to have distinct effects on the magnetic properties of NiPS_3_. Substituting just 9% Cr significantly suppresses magnetic anisotropy, and the field‐driven moment polarization‐like behavior can be observed under an external magnetic field of ≈8 T.^[^ ^26^ ^]^ In addition to metal substitutions, the non‐magnetic chalcogen substitution is also efficient in tuning magnetism in NiPS_3_. The SF transition for NiPS_3_ (x = 0) is pushed to a higher in‐plane field for x = 0.25 in NiPS_3‐x_Se_x_, which on further increasing Se content up to 1.3 results in essentially linear magnetization under both *H*//*ab* and *H*⊥*ab* fields up to 9 T.^[^ ^24^ ^]^ Unlike the case of metal substitutions where the mixing of two metal atoms creates frustration in the magnetic plane that consequently reduces the magnetic ordering temperature *T*_N_ with increasing substitution, the Se for S substitution enhances *T*_N_ because the magnetic exchanges occurring via superexchange interaction through chalcogen atoms become stronger owing to more extended orbital for Se as compared to S.^[^ ^24^ ^]^

In many MPX_3_ compounds including NiPS_3_,^[^ ^30^ ^]^ MnPS_3_,^[^ ^29^ ^]^ and FePS_3_,^[^ ^30^ ^]^ a broad hump in susceptibility occurs at temperature immediately above *T_N_* (Figure 7 in Ref.,^[^ ^30^ ^]^ Figure 3 in ref.[29] & Figure in 7 ref.[30]), which has been attributed to a short‐range 2D or quasi‐2D magnetic correlations owing to the layered structures. The broad hump in NiPS_3_ is found to be suppressed by metal Cr,^[^ ^26^ ^]^ Mn,^[^ ^29^, ^31^ ^]^ and Fe^[^ ^30^ ^]^ substitutions, which has been ascribed to the weakening of a short‐range magnetic correlation because of randomness introduced by substitution. In contrast, the Se substitution for S is relatively inefficient in modulating such short‐range magnetic correlations, which is manifested by the existence of broad hump in susceptibility accompanied by the clear difference between *T_N_* and broad maximum temperature *T*_max_ up to x = 1.3 in NiPS_3‐x_Se_x_.^[^ ^24^ ^]^ Such efficient tuning of magnetism is not only important for magnetic studies but can be fundamental for magneto‐optics given the strong coupling between magnetism and optically active excitons and phonons in NiPS_3_,^[^ ^5^, ^6^, ^14^ ^]^ which will be discussed below.

### 2.2 PL Measurements of SOX in Mixed MPX_3_ Compounds

**Figure 2:**
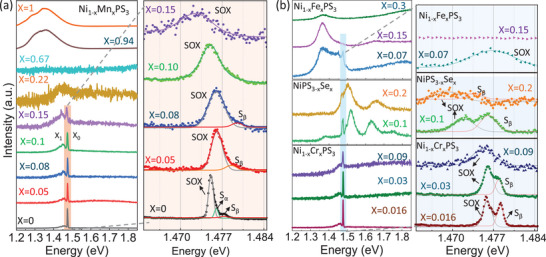
Low‐temperature PL spectra collected at 4.3K from Ni_1‐x_Mn_x_PS_3_ a), Ni_1‐x_Fe_x_PS_3_ (b, top), NiPS_3‐x_Se_x_ (b, middle), Ni_1‐x_Cr_x_PS_3_ (b, bottom) for x values indicated in the plot and zoom in view of the SOX emission at 1.476 eV (right panels).

**Figure 3:**
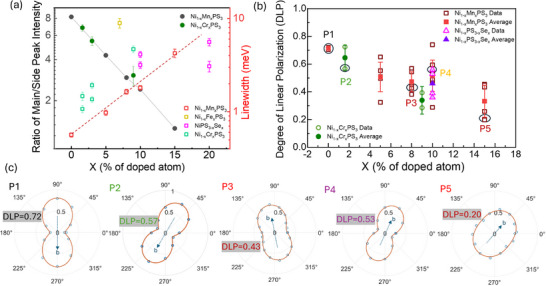
a) Left Axis: Normalized intensity of SOX emission (solid data points) for Ni_1‐x_Mn_x_PS_3_ (black) and Ni_1‐x_Cr_x_PS_3_ (green) plotted as a function of x. Right Axis: Linewidth of the SOX peak plotted as the function of x (open data points) for Ni_1‐x_Mn_x_PS_3_(red), Ni_1‐x_Cr_x_PS_3_ (green), Ni_1‐x_Fe_x_PS_3_ (yellow), and NiPS_3‐x_Se_x_ (magenta). b) Degree of linear polarization plotted against x for Ni_1‐x_Mn_x_PS_3_(red), Ni_1‐x_Cr_x_PS_3_ (green), and Ni_1‐x_Fe_x_PS_3_(yellow). c) Polar plots for the data points P1(NiPS_3_), P2 (Ni_0.984_Cr_0.016_PS_3_), P3 (Ni_0.92_Cr_0.08_PS_3_), P4 (NiPS_2.9_Se_0.1_) and P5 (Ni_0.85_Mn_0.15_PS_3_) indicated in (b) The b axes of the crystal have been indicated by dotted blue arrows.

**Figure** **2** summarizes the results of our low‐temperature (4.3 K) PL spectroscopy study on Ni_1‐x_Mn_x_PS_3_ (x = 0–1), Ni_1‐x_Fe_x_PS_3_ (x = 0–1), Ni_1‐x_Cr_x_PS_3_ (x = 0–0.09) and NiPS_3‐x_Se_x_ (x = 0–0.2). Overall, the data show the strong suppression of SOX emission (∼1.475 eV) and broadening of its linewidth with the increase of metal as well as chalcogen substitutions, similar to those reported in prior studies on Ni_1‐x_Cd_x_PS_3_^[^ ^32^ ^]^ and Ni_1‐x_Mn_x_PS_3_.^[^ ^33^ ^]^ However, a closer inspection of the data in Figure 2 reveals significant differences in the SOX emission depending on the metal and chalcogen substitutions. The SOX emission spectra of our pure‐phase NiPS_3_ can be fitted with three Gaussian peaks at 1.475, 1.476, and 1.477 eV with line widths of 0.5, 0.5, and 0.7 meV linewidths, respectively. These values are in qualitative agreement with those published earlier for the main SOX peak and two high energy shoulder peaks (S_α,_ S_β_) in NiPS_3_.^[^ ^5^, ^6^, ^7^, ^16^, ^17^ ^]^ Upon Mn substitution (Figure 2a), the SOX peak undergoes a spectral blue‐shift by ≈1.0 meV for an increase of x from 0 to 0.08 and then red‐shifts to lower energy (1.472 eV) with further increases in x to 0.22. While this trend of the shift in PL peak energy parallels the trend reported for Ni_1‐x_Cd_x_PS_3_,^[^ ^32^ ^]^ the prior study of Ni_1‐x_Mn_x_PS_3_ reported only the bule‐shift of the SOX peak with Mn substitution up to x = 0.1 and attributed to increase of bandgap from ≈1.5 eV of NiPS_3_ to 2.64 eV of MnPS_3_.^[^ ^33^ ^]^ The observation of red‐shift at higher x values indicate that this simple picture is not sufficient to explain the energy shift. The widths of the spectral lines (extracted via Gaussian line‐shape fitting) broaden exponentially with composition as indicated by linear variation on the semi‐log plot (**Figure** **3**a red squares, right axis). For x = 0.05, and 0.08, the peaks can be fitted with two gaussian peaks with the second peak at 1.478 and 1.479 eV respectively. These second PL peaks could evolve from the S_β_ peak of the pure NiPS_3_. The S_α_ peak on the other hand merges with the SOX peak due to spectral broadening. To quantify the loss of coherence and suppression in intensity and of the SOX we plot the linewidth and ratio of the SOX peak intensities to that of the maxima of the low energy side‐bands as functions of concentration x in Figure 3a (black solid circles, left axis and red open squares, right axis). The linear variation on the semi‐log plot observed on both plots again indicates the exponential suppression in intensity and coherence of the SOX emission with Mn substitution. While our study shows the persistence of the SOX emission up to x = 0.15 and complete quenching only at x = 0.22, the prior study (ref.[33]) of Ni_1‐x_Mn_x_PS_3_ reported the supression of the SOX emission for Mn content up to x = 0.1. Even at this concentration, a broadened SOX peak is still clearly visible in their data. Our study extend the composition boundary for SOX suppression. Additionally, it is worthy to note that all compositions in this work were rigorously verified through multiple EDS scans to ensure accuracy of composition and homogenity in all single crystal samples.

A similar exponential decrease of SOX emission is also observed with Cr substitution as evidenced by the plot of the SOX/side‐band intensity ratio (solid green data points in Figure 3a). In contrast to a simple broadening of the spectral line reported for Cd and Mn substitutions,^[^ ^32^, ^33^ ^]^ the high‐resolution PL spectra of Ni_1‐x_Cr_x_PS_3_ (Figure 2b bottom 3 panels) reveal a significantly different spectral evolution. Two sharp PL peaks at 1.476 and 1.478 eV are observed for x = 0.016 with similar intensities and linewidths (1.47 and 1.07 meV, respectively). Based on the energy we attribute the two peaks to SOX and S_β_. While the SOX peak undergoes a blue‐shift in energy to 1.476 eV, the S_β_ peak red‐shifts to 1.478 eV for x = 0.03. The linewidths of both peaks increase to 1.92 and 1.36 meV, respectively. The two peaks merge into a single broad peak at x = 0.09 with a peak energy of 1.475 eV and linewidth of 4.58 meV. This trend of S_β_ peak strengthening and merging toward the SOX peak with the increase of Cr substitution stands in contrast to the behavior of the S_β_ peak in Ni_1‐x_Mn_x_PS_3_. On the other hand, the SOX peak undergoes an energy blue‐shift and red‐shift as in the case of Cd and Mn substitutions. This behavior of S_β_ peak further suggests that substitution of the transition metals could lead to the modification of the excitonic fine structure of the SOX state and more studies to identify the origin of the S_α_ and S_β_ necessary.

The doublet emission feature is also observed in the case of NiPS_2.9_Se_0.1_ at the peak energies of 1.472 and 1.476 eV with linewidths of 4.12 and 3.17 meV, respectively. Both peaks red‐shift to 1.4685 and 1.473 eV and broaden to 5.59 and 3.07 meV when x is increased to 0.2. In addition to the SOX emission, two broad emission bands at ∼1.52 and 1.63 eV are also observed. The 1.52 eV broad‐band red‐shifts to merge with the SOX emission at x = 0.2, making the study of SOX for x > 0.2 impossible. Since these broad bands were not observed in the cases of transition metal substitution, we tentatively attribute them to emissive defect states resulted from Se substitutions. Finally, in the case of Ni_1‐x_Fe_x_PS_3_ (x = 0–1), we observed a near‐complete suppression of the SOX peak at the lowest x value of 0.07. The SOX peak only appears as a very small sharp spike on the high energy shoulder of a broad peak centering at 1.35 eV. The Gaussian fit to this sharp spike (Figure 2b) yields a FWHM of 8.91 meV (the yellow data point in Figure 3a). At x ≥ 0.15 the sharp spike completely disappears and only the broad 1.35 eV peak is visible. This peak undergoes a bule‐shift in energy and becomes weaker with increasing x. We attribute this low energy broad peak to the electronic Raman scattering corresponding to d‐d transitions.^[^ ^46^ ^]^ At the 2.33 eV laser excitation, the electronic Raman peak which is reported to appear at a 1.0 eV energy shift from the laser excitation energy happen to appear at the emission energy just below the SOX emission.^[^ ^46^ ^]^ When the laser excitation energy is tuned from 2.33 to 3.06 eV, this peak moves with laser energy maintaining the 1.0 eV energy shift, confirming the assignment (Figure S1, Supporting Information).

We also conducted polarization‐resolved PL spectroscopy to analyze the anisotropy of the SOX emission. Figure 3b plots the degree of linear polarization DLP = (I_S_ – I_P_)/ (I_S_ + I_P_), where I_S/P_ correspond to the intensity of S or P polarized emission, measured for Ni_1−x_Mn_x_PS_3_, Ni_1−x_Cr_x_PS_3,_ and NiPS_3−x_Se_x_ as a function of x. The sample polar plots of I_S_/(I_S_ + I_P_) for different data points in Figure 3b (labeled P1‐P4) are displayed in Figure 3c. Consistent with prior studies, the SOX of pure NiPS_3_ displays a strong polarization anisotropy with DLP = 0.74. Almost all the pure NiPS_3_ flakes investigated display very little variation in DLP (P1 in Figure 3b,c). On the other hand, the DLP values vary significantly for the alloyed samples (different data points at each x values represent measurements on different flakes) and the average DLP values decreases to 0.381 ± 0.062 at x = 0.15. A similar decrease of DLP is also observed for introduction of Cr and Fe substituents. The decrease in DLP values indicates the increased disorder in XXZ‐type AFM of NiPS_3_ with substitutions as intended. The increase in spread of DLP values on the other hand indicates the position dependent variation in disorder which is unavoidable.

These findings together reveal that all the key characteristics SOX (intensity, linewidth, energy, and DLP) can be strongly modified in parallel by the introudction of substituents. We tentatively attributed these changes as a consequence of change in formation, binding energy, and coherence of the SOX exciton state resulted from the disturbance to the magnetic anisotropy, spin orientation and metal‐sulfur charge transfer process induced by the substituents. A direct comparison of our findings, on the other hand, indicates that there are significant quantitative differences across the different types of the substituents. These differences are collrelated with the mangetic properties reported in prior works and Raman studies of the next session in the Discussion section.

### 2.3 Raman Spectroscopy of Mixed MPX_3_ Compounds

**Figure 4:**
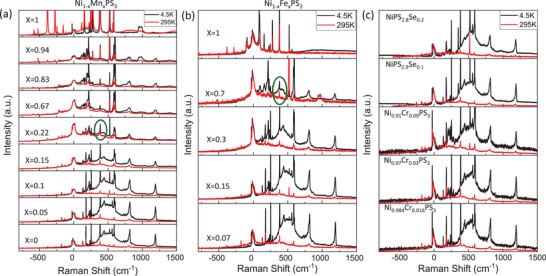
Raman spectra of Ni_1‐x_Mn_x_PS_3_a), Ni_1‐x_Fe_x_PS_3_b), NiPS_3‐x_Se_x_ c, top) and Ni_1‐x_Cr_x_S_3_(c, bottom) acquired at 4.5 K (black spectra) and 295 K (red spectra). The peaks around 520 and 950 cm^−1^ in some spectra correspond to the silicon substrate.

**Figure 5:**
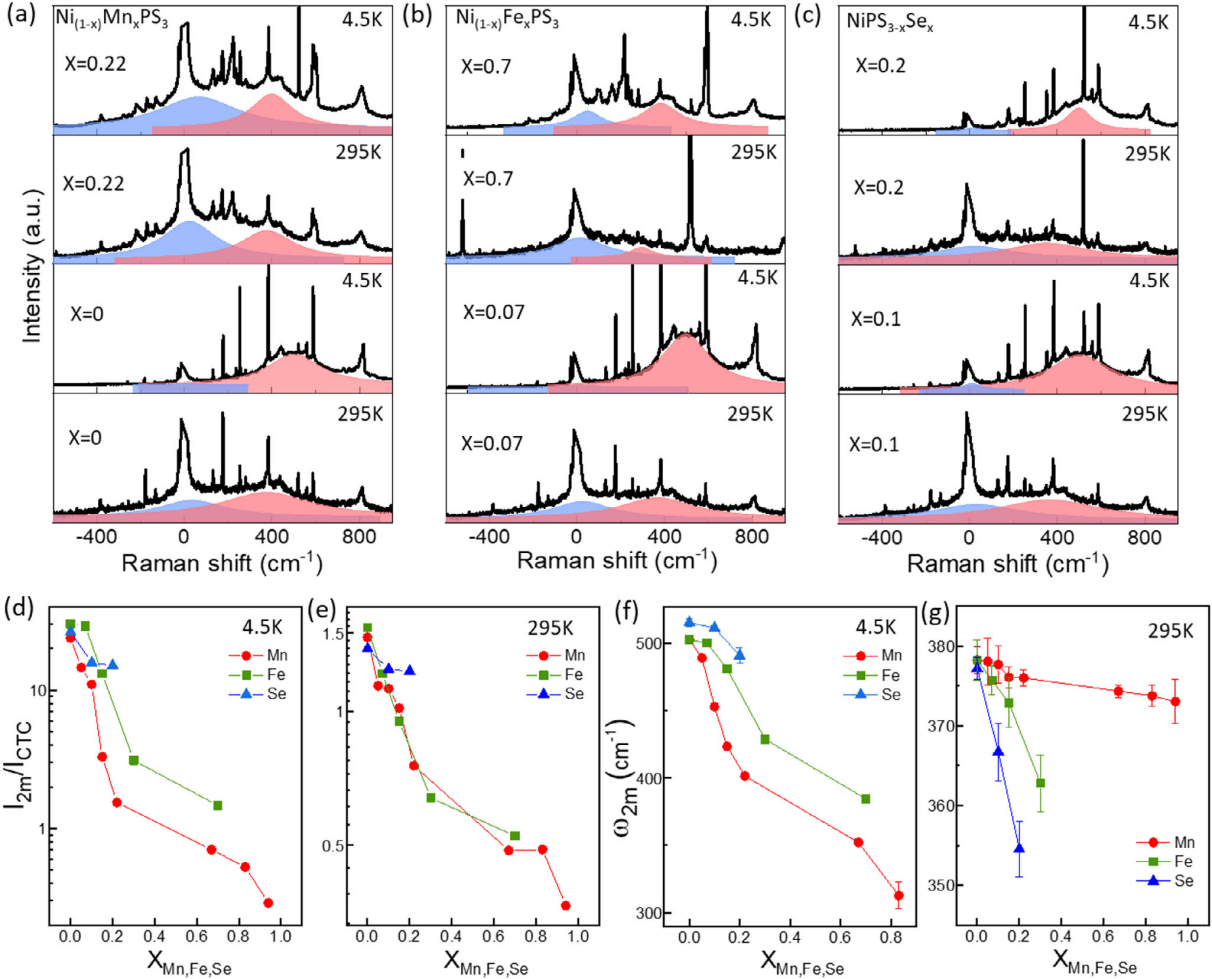
Raman spectra at 4.5 and 295K for Ni_1‐x_Mn_x_PS_3_ with x = 0, 0.22a), Ni_1‐x_Fe_x_PS_3_ with x = 0.07,0.7 b) and NiPS_3‐x_Se_x_ with x = 0.1, 0.2c). The two continua CTC and 2M  are shown under each spectrum in blue and red, respectively (the fit data for complete set of Raman spectra are shown in Figures S2 and S3 (Supporting Information). d,e) Ratio of Raman intensities between the 2M  and CTC peaks (I_2M_/I_CTC_) at 4.5 K d) and 295 K e) extracted from the fitted continuum are plotted as functions of x for Ni_1−x_Fe_x_PS_3_ (red circles), Ni_1−x_Mn_x_PS_3_ (green squares) and NiPS_3−x_Se_x_ (blue triangles). f,g) Plots of 2 m peak frequency (ω_2 m_) vs x at 4.5 K f) and at 295 K (g).

Next, we turn to Raman spectroscopy studies of the alloyed NiPS_3_ compounds. **Figure** **4**a–c compare the low temperature (4.5 K, black spectra) and room temperature (295K, red spectra) unpolarized Raman spectra collected with 532 nm excitation from Ni_1‐x_Mn_x_PS_3_, Ni_1‐x_Fe_x_PS_3_, Ni_1‐x_Cr_x_PS_3_, and NiPSe_x_S_3‐x_ for various substituent concentrations. Several interesting changes can be observed in the spectra with the addition of substituents. New peaks emerge in the Raman spectra of NiPS_3_ with increasing substitutions. Moreover, one can clearly see the background underneath the spectra move with increasing substituent content. This is because of dramatic changes in the two continua underlying the peaks in the Raman spectra. The two continua are displayed more clearly as red and blue shaded curves for a few select substituent concentrations in **Figure** **5**a–c, with the fitted curves for all the compositions at 4.5 and 295 K provided in Figure S2 (Supporting Information). The examples of curve fitting from original spectra are shown in Figure S3 (Supporting Information). The high‐frequency continua ∼500 cm^−1^ (red peaks in Figure 5a–c; Figure S2, Supporting Information) are attributed to 2M  scattering,^[^ ^39^, ^47^, ^48^ ^]^ where the incident light is scattered by two magnons with equal and opposite wavevectors, similar to a second order two‐phonon scattering process. As mentioned above, this peak can be observed even in the room temperature paramagnetic state due to short‐range correlations in the magnetic sub‐lattice in NiPS_3_. The second feature at ∼35 cm^−1^ is attributed to the CTC (blue peaks in Figure 5a–c; Figure S2, Supporting Information). As mentioned above, NiPS_3_ is a well‐known negative charge transfer insulator wherein the proximity of the S atoms to the Ni^2+^ ions^[^ ^9^ ^]^ enables the transfer of electrons from S to Ni^2+^.^[^ ^49^ ^]^ This charge transfer is likely responsible for the appearance of the low‐frequency CTC in the Raman spectrum of NiPS_3_, as supported by another recent Raman study on Ni_1‐x_Zn_x_PS_3_.^[^ ^41^ ^]^ We note that the CTC peak is different from the quasielastic scattering (QES) peak also observed in NiPS_3_.^[^ ^9^, ^38^ ^]^ The QES occurs from fluctuations of the energy density of the spins and is only observable at room temperature in the orbitally disordered paramagnetic state.^[^ ^50^ ^]^ It manifests in the Raman spectrum as a rising background centered at 0 cm^−1^,^[^ ^9^ ^]^ and appears in addition to the CTC peak, which occurs at higher frequencies ∼35 cm^−1^. The CTC and 2M  contributions can be observed in both the room and low‐temperature Raman spectra of all mixed MPX_3_ compounds but their relative contributions to the spectra vary significantly with the type and concentration of the substituent.

The intensity of the CTC peak increases with substituent concentrations, although it is hard to quantify this increase considering the numerous changes in all the Raman peak intensities as well as the appearance of new peaks with increasing substituent concentrations. Nevertheless, we show the intensity of the CTC peak relative to the 380 cm^−1^ peak in Figure S4 (Supporting Information). The 380 cm^−1^ peak is attributed to out‐of‐plane stretching (A_g_ symmetry) of the chalcogen atoms and is common to all the MPS_3_ compounds.^[^ ^51^ ^]^ While the CTC peak intensity increases relative to the 380 cm^−1^ peak with increasing Fe, Mn, and Se content (Figure S4, Supporting Information), we can only consider this intensity ratio for low concentrations (up to x = 0.22 for Mn and x = 0.3 for Fe) since the intensity of the 380 cm^−1^ mode is also severely modulated at higher concentrations. However, a definite and significant effect of alloying at room temperature is a sharp decrease in the intensity of the 2M  peak with increasing Mn/Fe/Se contents. This can be seen more clearly in Figure 5d,e, which plots the intensities of the 2 m peaks relative to the CTC peaks (I_2M_/I_CTC_) as functions of x for Fe, Mn, and Se substitution as a function of x at 4.5 and 295K, respectively. While the decrease of I_2M_/I_CTC_ is nearly identical for Fe and Mn substituents, it is slower for the case of Se substitution (blue data in Figure 5a). This suggests that the short‐range correlations are stronger between the metal ions and S ligands compared to Se. At low temperature (4.5 K), the 2M  peak also exhibits an energy red‐shift in addition to the suppression of its intensity, and it displays significant differences between different types of substituents, especially in the case of Mn and Fe. For Mn substitution, the energy of the 2M  peak red‐shifts from 500 cm^−1^ at x = 0 to 400 cm^−1^ at x = 0.22 (Figure 5e) and its intensity is suppressed to the point that Raman spectra at 4.5 K (below T_N_) becomes almost identical (green circle in Figure 4a) to that at RT (above T_N_). In contrast, the red‐shift and intensity decrease of 2M  peak at 4.5K occurs at a slower rate for Fe substitution such that a distinct 2M  peak is observable in the 4.5 K Raman spectrum up to x = 0.7 (Green circle in Figure 4b). While the introduction of both Cr and Se substituents also leads to similar red‐shifts and decrease in intensity, the 2M  peak can be observed up to the highest available Cr and Se contents of x = 0.09 and x = 0.2, respectively (Figure 4c).

Unfortunately, we were unable to resolve the CTC peak in the Raman spectra from the Cr‐substituted NiPS_3_, so we excluded these data from the analysis of Figure 5. Furthermore, we only include data for Mn and Fe substitutions up to x = 0.22 and x = 0.7, respectively, because at higher concentrations, the Raman spectrum changes dramatically with the emergence of new peaks, redshifted peak frequencies, and the gradual disappearance of the NiPS_3_ Raman peaks. These changes in the Raman spectra are accompanied by lattice distortions and bond lengthening in the mixed MPS_3_ compounds. As was shown in Ref. [9] NiPS_3_ has the smallest unit cell owing to its short M‐S bond distance, followed by FePS_3_ and MnPS_3_. It follows that the accommodation of Fe and Mn into the NiPS_3_ lattice would lead to bond lengthening, and hence red‐shifted Raman peak frequencies as well as disruptions to the charge transfer between the Ni^2+^ ions and the S/Se atoms. Figure S5 (Supporting Information) shows the redshifted phonon mode frequencies due to lattice distortions in the mixed NiPS_3_ compounds. The other observation from Figure 4 is that, in addition to the 2M  peak, all the Raman peaks also decrease in intensity with increasing Fe or Mn substitution. However, the CTC peak persists relative to these peaks, suggesting that the charge transfer between the metal ions and S ligands remains strong even with metal ion substitution. This can be seen more clearly in Figure S2 (Supporting Information) where the CTC peak remains up to x = 0.94 and 0.7 in Ni_1‐x_Mn_x_PS_3_ and Ni_1‐x_Fe_x_PS_3_, respectively.

**Figure 6:**
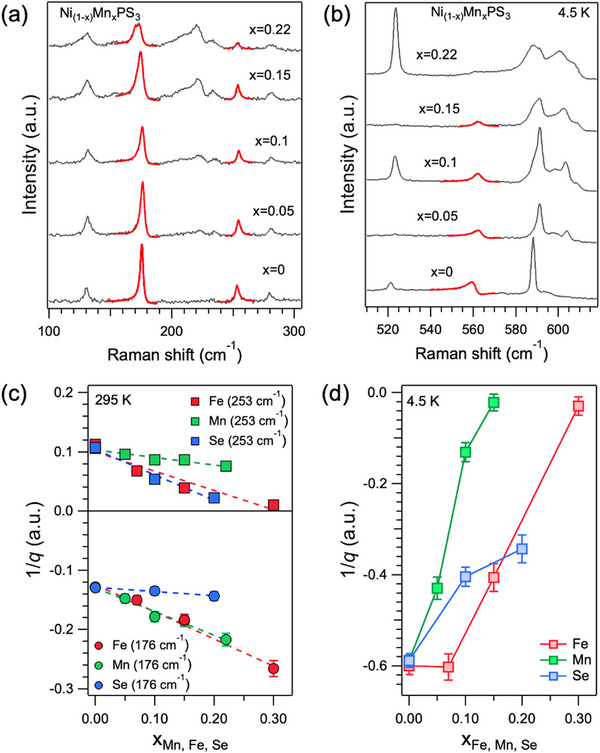
Raman spectra from Ni_1‐x_Mn_x_PS_3_ for x = 0 to 0.22 at a) 295 K and b) 4.5 K. The asymmetric peaks ∼176 and 253 cm^−1^ have been fit with BWF lineshapes (red) and overlaid on top of the raw spectra. c) Fano parameters (1/q) at 295 K as a function of substituent concentration (Mn, Fe, and Se) for the 253 cm^−1^ (top panel) and 176 cm^−1^ mode (bottom panel). d) Fano parameters (1/*q*) at 4.5 K as a function of substituent concentration (Mn, Fe, and Se) for the high frequency 550 cm^−1^ mode.

Next, we discuss the effects of the substitutions on electron phonon coupling (EPC). As mentioned above, there are three Raman modes in NiPS_3_ that exhibit asymmetric Fano line‐shapes because of EPC. Two vibrational modes (∼176 and 253 cm^−1^) are asymmetric at room temperature while a high frequency mode ≈560 cm^−1^ is asymmetric in the AFM state. The symmetry of the 176 cm^−1^ peak is E_g_ (obtained from polarized Raman spectroscopy measurements),^[^ ^51^ ^]^ and corresponds mainly to in‐plane vibrations of the S and P atoms with a minor contribution from the Ni atoms. The 253 cm^−1^ peak corresponds to out‐of‐plane vibrations of the S atoms (A_g_ symmetry) and the 560 cm^−1^ peak corresponds to in‐plane symmetric and anti‐symmetric vibrations of the S and P atoms. We note that while the 176 cm^−1^ peak appears as a single asymmetric peak at room temperature, it splits into two peaks below the AFM transition temperature and can be observed through polarized Raman measurements.^[^ ^38^ ^]^ However, the splitting is absent at room temperature and we can obtain insights into the EPC by studying the room temperature asymmetric Fano lineshape. **Figure** **6**a shows room temperature Raman spectra in the frequency range between 100 and 300 cm^−1^ from Ni_1‐x_Mn_x_PS_3_ for x up to 0.22. The two modes exhibiting Fano lineshapes ∼176 and 253 cm^−1^ have been fit with Breit‐Wigner Fano (BWF) lineshapes (the red fits are shown on top of the raw spectra) according to the formula

(1) $$I\left(\omega \right)={\left[1+\frac{2\left(\omega_{0}-\omega \right)}{q\Gamma }\right]}^{2}/\left[1+\frac{4{\left(\omega_{0}-\omega \right)}^{2}}{\Gamma^{2}}\right]$$

where ω_0_, Γ, and *q* are the phonon mode frequency, peak width, and asymmetry parameter, respectively.^[^ ^52^ ^]^ The reciprocal of this last term, 1/*q* is the Fano parameter, which represents the degree of asymmetry and thus the strength of the EPC. When 1/*q* = 0, the peak becomes symmetric with a Lorentzian lineshape. The high‐frequency Fano peak 560 cm^−1^ (collected at 4.5 K) is similarly plotted in Figure 6b. We note that the asymmetry of the three peaks is not the same – the lowest and highest frequency modes (176 and 560 cm^−1^, respectively) have asymmetric tails on their low‐frequency sides while the 253 cm^−1^ peak is asymmetric on its high frequency side. These differences are due to the phonon modes coupling with electronic continua on their low or high frequency sides, and result in positive and negative Fano parameters for the 176/560 and 253 cm^−1^ peaks, respectively (the 176 cm^−1^ phonon mode mainly couples to the CTC continuum at room temperature, while the 253 and 560 cm^−1^ peaks couple to the 2M  continuum at room temperature and below T_N_ respectively). The Fano parameters obtained from the room temperature Raman spectra are plotted as functions of x in Figure 6c, and exhibit contrasting trends. The parameter for the 176 cm^−1^ peak becomes more negative (i.e., the peak becomes more asymmetric) with x going from 0 to 0.22, while the Fano parameter for the 253 cm^−1^ peak decreases closer to 0 (peak becomes more symmetric). The decrease in 1/*q* for the 176 cm^−1^ mode is because of increased EPC. This can be rationalized by the low frequency asymmetric tail of the 176 cm^−1^ peak, which couples to the CTC, hence the increasing EPC for this mode with substitutional doping. On the other hand, the high frequency tail of the 253 cm^−1^ mode occurs due to its coupling with the 2M  continuum, which diminishes with increasing Mn content (as shown in Figure 4a). Thus, the 253 cm^−1^ mode becomes more symmetric, and its EPC decreases with doping. At low temperature, the high‐frequency 560 cm^−1^ peak becomes more symmetric (1/q goes closer to 0) with increasing Mn content up to x = 0.22. This is concomitant with the decrease in intensity of the 2M  peak with substitutional doping (Figure 4d). The above results show that, with increasing substituent content, the room temperature EPC of the 176 cm^−1^ peak increases while it decreases for the other two peaks. Remarkably, even though the asymmetry of this peak increases with substitutional doping at room temperature, its behavior mirrors that of the CTC and SOX at low temperatures. The relatively weak response of the EPC to Se substitution (Figure 6b,c) compared to Fe or Mn substitution is concurrent with the indifference of the 2M  and CTC peaks to Se substitution (Figure 5a). We also see broadening of other Raman peaks with increasing substituent concentrations, as expected. We analyzed the linewidths of the 380 cm^−1^ (Figure S6, Supporting Information), which increase with Fe, Mn, and Se concentrations. Not surprisingly, this increase is the highest for NiPS_3‐x_Se_x_, where Se substitution affects the chalcogen vibrations more than metal ion substitution. These results highlight the importance of the charge transfer between the metal ions and the S ligands, which, upon substitutional doping by Fe^2+^ and Mn^2+^ ions, seemingly affect EPC at room temperature (especially for the in‐plane phonon mode ∼176 cm^−1^) and the SOX emission at low temperatures.

## 3 Discussion

A direct comparison among substitution‐dependent evolutions of SOX (PL), CTC, and 2M  (Raman) peaks reveals several intriguing behaviors that differ markedly with the type of substituent. The microscopic origin of SOX emission in NiPS_3_ has been debated.^[^ ^5^, ^6^, ^16^, ^18^, ^19^, ^20^, ^21^, ^23^ ^]^ As shown below, among the different substituents, Fe and Mn substitutions produce the most pronounced contrast in the evolution of the SOX and Raman features. This contrast is consistent with the predicted strong coupling between AFM structure and SOX.^[^ ^18^ ^]^ In NiPS_3_, each Ni^2+^ ion in a distort octahedral crystal field possesses a high spin ground state with half‐filled e_g_ orbitals. SOX is predicted to corresond to an onsite spin‐flip d‐d multiplet transition that locally converts this S = 1 ground state to an S = 0 excited state, producing a sharp PL resonance (i.e., SOX) near 1.47 eV. Due to such coupling with spins, SOX is expected to be sensitive to the magnetic structure and moment orientations (magnetic anisotropy) of NiPS_3_. In our Fe‐substituted samples, the SOX peak is strongly suppressed and nearly disappears at the lowest substitution content x = 0.07 (Figure 2b). This observation is reminiscent of the turnover from the XXZ to Ising anisotropy at light Fe‐substitution in Ni_1‐x_Fe_x_PS_3_, which was previously identified by magnetization^[^ ^30^ ^]^ and terahertz spectroscopy.^[^ ^53^ ^]^ Although theory predicts a critical composition of x = 0.02,^[^ ^53^ ^]^ experimental reports determined the onset of x = 0.1,^[^ ^30^, ^53^ ^]^ consistent with the critical composition of 0.07 probed in this study. Given that the XXZ‐type magnetism in NiPS_3_ is characterized by primarily in‐plane spins with rather weak out‐of‐plane spin component, we conclude that XXZ anisotropy, especially its in‐plane anisotropy, play an essential role in SOX emission in NiPS_3_, consistent with the theoretical prediction described above.^[^ ^18^ ^]^

On the other hand, the 2M  peak exhibits a slow suppression with substituent concentration such that a distinct 2M  peak is visible up to x = 0.7 in the 4.5 K Raman spectra (Figure 4b). A similar slow suppression of 2M  scattering is also reported in ref. [42] and attributed to the persistence of short‐range magnetic correlations after the turnover from XXZ to Ising anisotropy at x = 0.1. This finding further suggests that the short‐range magnetic correlation may play a lesser role in the SOX emission while they are critical for 2M m scattering. This short‐range correlation is gradually suppressed by the enhanced magnetic fluctuations/frustrations arising from the mixing of distinct magnetic lattices of MPX_3_, leading to slow suppression of 2 m scattering by substitution.

The correlations between SOX emission and magnetic anisotropy as well as between 2M  scattering and short‐range correlations also explains the observation of Mn substitution. Unlike Fe substitution, in Mn‐substituted Ni_1‐x_Mn_x_PS_3_, both SOX emission and 2M  scattering at 4.5 K are suppressed with increasing x in similar ways such that both peaks essentially disappear at x = 0.22. These findings imply that Mn substitution suppresses short‐range magnetic correlations at the same strength as changing magnetic anisotropy. For magnetic anisotropy that is correlated with SOX, the change in magnetic anisotropy occurs from XXZ with nearly in‐plane spins in NiPS_3_ to Néel type ordering with out‐of‐plane spin in MnPS_3_ (see magnetic structures in Figure 1). The in‐plane spins of NiPS_3_ rotate gradually with the introduction of Mn substitution to finally yield an out‐of‐plane easy axis of MnPS_3_. As discussed in Section 2.1, the strong suppression of the SF transition in NiPS_3_ by 5 % Mn substitution indicates easy axis rotation starting from x < 0.05.^[^ ^28^ ^]^ Further study suggests a significant easy axis rotation to the out‐of‐plane direction around x = 0.5.^[^ ^29^ ^]^ These upper and lower bound of x values for the spin turnover further corroborate that disturbances in XXZ magnetic anisotropy play a critical role in the strong suppression of the SOX peak that starts immediately at the lowest value of x = 0.05 and reaches completion at x = 0.22 in our samples. The disturbance in XXZ magnetic anisotropy and spin orientation as well as the associated suppression of SOX induced by Mn substitutions appear to be significantly weaker than that induced by Fe substitutions. This can be understood by the fact that Fe^2+^ has extremely strong anisotropy because its high spin *d* ^6^ state and unquenched orbital angular momentum due to partially filled t_2g_ orbitals enhance spin‐orbit coupling, which leads to strong modification of magnetic anisotropy up on Fe substitution as stated above. However, half‐filled *d*‐orbital (*d* ^5^) of Mn^2+^ leads to vanishing orbital angular momentum and spin‐orbit coupling similar to the Ni^2+^ ions with fully filled *t*_2g_ and two electrons in *e*_g_ orbitals. The much weaker single ion anisotropy of Mn^2+^ leads to less efficient suppression of SOX in Mn‐substituted NiPS_3_ as compared to Fe‐substitution.

Regarding the impact of Mn‐substitution on 2M  scattering and short‐range correlations, as reviewed in the previous Section 2.1, temperature‐dependent magnetic susceptibility of MPX_3_ compounds revealed that short‐range 2D or quasi‐2D magnetic correlations of these materials manifest as a broad maximum in susceptibility at a temperature T_max_ above T_N_. In the case of Ni_1‐x_Mn_x_PS_3_, T_max_ and T_N_ converge to essentially the same value when x is increased from 0 to 0.3 (Figure 8 in ref. [31]) indicating that short range correlations is significantly suppressed below 30% Mn substitution, in agreement with our observation of suppression of 2 m scattering at x = 0.22. Therefore, compared to Fe substitution where the 2M  peak is still visible at x = 0.7, Mn substitution suppresses short range correlations more efficiently. This is also why the CTC peak persists up to x = 0.22 in Ni_1‐x_Mn_x_PS_3_ (Figure 4a), considering that the CTC corresponds to long‐range charge transfer between the metal ions and chalcogen atoms. These trends can be understood in terms of the different exchange interactions of these magnetic ions in MPX_3_. In MPX_3_ compounds, while the strength of exchange interactions of Fe^2+^ and Ni^2+^ are relatively comparable, that of the Mn^2+^ is much weaker.^[^ ^27^ ^]^ Additionally, magnetism is dominated by super‐exchange in FePS_3_ and NiPS_3_ but by direct exchange in MnPS_3_.^[^ ^27^ ^]^ Such distinction of Mn^2+^ as compared to Fe^2+^ and Ni^2+^ implies that Mn^2+^ substitution in NiPS_3_ system can induce a strong disorder in magnetism. Therefore, the short‐range correlations in NiPS_3_ and related 2M  scattering is relatively robust against Fe substitution, but is quickly suppressed with low Mn substitution. Such a scenario, i.e., the correlation between 2M  scattering and magnetic exchange interactions in NiPS_3_, is further supported by our observations in chalcogen‐substituted NiPSe_x_S_3‐x_. The nature of magnetism in NiPS_3_ is governed by Ni─S─Ni superexchange, which is enhanced by Se substitution due to more extended 4*p*‐orbital of Se than 3*p*‐orital for S.^[^ ^21^, ^24^ ^]^ Therefore, 2M  scattering is robust against Se substitution as shown in Figure 5a, as well as the lower slopes in the Raman data in Figure 6.

Cr substitution induces a similar degree of suppression in both SOX emissions compared to the case of Mn substitution. The 2M  scattering peak can also be observed for all Cr contents up to 0.09. However, because the CTC continuum does not appear clearly in the Raman spectrum, we could not compare the relative strength (i.e., I_2M_/I_CTC_) as in the case of other substituents. These trends suggest that Cr substitution induces a disturbance to the magnetic anisotropy and short‐range correlation approximately at the same degree as Mn substitution. Indeed, in Ni_1‐x_Cr_x_PS_3_, Cr substitution for Ni has been demonstrated to reduce the magnetic anisotropy^[^ ^26^ ^]^ similar to that of Mn substitution.^[^ ^27^ ^]^ Also, though CrPS_3_ has not been reported yet, the study on related CrPSe_3_ revealed that the nature of magnetic exchange interaction for Cr^2+^ is dominated by the nearest neighboring interacting J_1_,^[^ ^54^ ^]^ similar to that of Mn^2+^.^[^ ^27^ ^]^ Moreover, the appearance of two distinct SOX peaks and their concentration‐dependent evolution (Figure 2c) indicate that Cr substitution can also strongly influence the electronic fine structure and optical selection rule of the SOX state. The appearance of a similar SOX doublet and concentration‐dependent evolution as well as suppression of 2M  scattering in the case of NiPSe_x_S_3‐x_ also indicates that the chalcogen atoms also have an influence on the spin orientation and short‐range correlations of the magnetic Ni^2+^ ions, but not as much as those produced by metal ion substituents. Studies of the SF transition and broad maximum in temperature‐dependent susceptibility^[^ ^24^ ^]^ corroborate that XXZ‐type magnetism and short‐range correlations, while being suppressed by Se substitution, should still be present at x = 0.2 in agreement with our results.

## 4 Conclusion

Our study presents a comprehensive analysis on the evolution of SOX emission, 2M  scattering processes, metal‐sulfur charge transfer and EPC in single‐crystalline NiPS_3_ as functions of concentration and type of substituents. The comparison study of the impact of various substituents allows for clarifying the governing factors of SOX and 2 m scattering. While both SOX emission and 2M  scattering are characteristics of the correlated antiferromagnetism in NiPS_3_, our work revealed that they are dictated by two distinct magnetic properties: 1) magnetic anisotropy, spin orientation, and metal‐sulfur charge transfer controlling SOX emission and 2) short‐range correlations dictating 2M  scattering. Due to extremely strong anisotropy derived from the high spin state, the introduction of Fe^2+^ ions leads to the strongest suppression of XXZ antiferromagnetism and SOX emission. And despite having exchange interactions and ionic radii comparable to those of Ni, Fe substituents give the weakest suppression of 2M  scattering. On the other hand, the significantly smaller exchange interaction of Mn substituents give rise to the strongest suppression of 2M m scattering compared to all other substituents, and at the same time, the reduced spin‐orbit coupling of Mn^2+^ also leads to weaker suppression of XXZ antiferromagnetism and SOX emission. The mechanism can be extended to Cr substituted NiPS_3_, which displays similar suppression of XXZ antiferromagnetism and SOX emission like the Mn substitution. These findings together revealed the introduction of substituents as the powerful route to control the emergent collective excitations of MPX_3_ materials.

## 5 Experimental Section

### Sample Growth and Characterization

All the single crystals used in this work were synthesized by a chemical vapor transport (CVT) method using I_2_ as the transport agent. For each composition except for pristine MnPS_3_, elemental powders with desired ratios were sealed in an evacuated quartz tube and heated in a two‐zone furnace with a temperature gradient from 750 to 550 °C for a week. For pristine MnPS_3_, it was synthesized using a slightly lower temperature gradient from 650 to 550 °C. The CVT growth yielded large millimeter‐sized plate‐like single crystals for all the compositions. Our previous reports have confirmed the phase, successful substitution, and excellent crystallinity through X‐ray diffraction (XRD) for Ni_1‐x_Mn_x_PS_3_,^[^ ^28^ ^]^ Ni_1‐x_Cr_x_PS_3_,^[^ ^26^ ^]^ and NiP(S_3‐x_Se_x_).^[^ ^24^ ^]^ For Ni_1‐x_Fe_x_PS_3_, XRD data are provided in Figure S6 (Supporting Information). The elemental compositions of the obtained crystals were examined by energy dispersive X‐ray spectroscopy (EDS). To ensure composition homogeneity, the final composition was confirmed by performing the EDS scan on multiple locations of each crystal. The compositions reported in this work are actual compositions determined by EDS, as summarized in Table S1 (Supporting Information). Flakes for measurements were exfoliated from the obtained bulk crystals using blue tape onto a commercial Si substrate with 285 nm SiO_2_ on top. Thick and uniform flakes (bulk‐like) were chosen for the optical measurements.

### Low‐Temperature Photoluminescence Spectroscopy

PL spectroscopy measurements on thick exfoliated flakes were performed at 4 K through a continuous Helium flow cryostat (Oxford Instruments, MicrostatHiRes). For optical excitation, a 532 nm continuous wave laser was used. A diffraction limited laser spot can be achieved using 40× microscope objective with a numerical aperture of 0.7. A lens is put before the objective in the excitation path to expand the laser spot for wide‐field measurements, and simply removing the lens will change the setup to confocal mode. The spectrum emission from the sample was collected through the same microscope objective and long‐pass filters, dispersed with either 150 or 600 g mm^−1^ grating (Action SP2300i), and measured by a liquid nitrogen‐cooled silicon charge coupled device camera (Roper Scientific Pylon) for PL spectroscopy. For the linear polarization test, a half‐wave plate and a Wollaston prism were used together to put into the detection path. The PL spectra were acquired as a function of the rotation angles of half‐wave plates to determine the degree and orientation of emission's linear polarization.

### Raman Spectroscopy measurements

The Raman Spectroscopic measurements were conducted through Horiba Lab RAM HR high evolution confocal Raman microscope. Samples were measured in both cryogenic and room temperature conditions in reflection mode using a 532 nm continuous wave laser (Oxxius LCX‐532S‐100) as excitation, inside of a flow cryostat (Oxford Instruments, MicrostatHiRes). The experiment was configured using a 2400mm^−1^ holographic grating blazed at 500 nm, a 150 µm confocal hole diameter with a 50×, 0.7 NA, cover glass‐corrected microscope objective (Olympus LCPLFLN50XLCD). Spectral calibration was performed using the 1332.5cm^−1^ band of a synthetic type II diamond.^[^ ^55^ ^]^ The spectral intensity was calibrated through a VIS‐halogen light source (NIST test no. 685/289682‐17).

## Conflict of Interest

The authors declare no conflict of interest.

## Author Contributions

C.T.T. and N.L. contributed equally to this work. H.H. and J.H. conceived the experiment. C.T.T. and N.L. conducted photoluminescence and Raman spectroscopy measurements for Ni_1‐x_Mn_x_PS_3_, Ni_1‐x_Fe_x_PS_3_, NiPS_3‐x_Se_x,_ and Ni_1‐x_Cr_x_PS_3_. All Raman measurements were carried out with assistance from R.K. and M.T.P. V.C, and A.C.J provided support for the initial PL setup and measurements performed by C.T.T. and N.L. N.L. performed analysis of PL spectral data. R.R. performed analysis and interpretation of the Raman spectral data. R.B. and D.U. performed crystal growth and characterizations. J.H., H.H., and R.R. wrote the manuscript with the assistance of all authors. All authors have given approval to the final version of the manuscript.

## Supporting information



Supporting Information

## Data Availability

The data that support the findings of this study are available from the corresponding author upon reasonable request.
