# Supplementary material for: Manipulation of Emergent Collective Excitations via Composition Control in Mixed MPX3 Correlated 2D Antiferromagnets
Source: Adv Sci (Weinh). 2025 Dec 12;13(12):e17378. doi: 10.1002/advs.202517378 (PMC12948200; doi:10.1002/advs.202517378)
Supplement: Supplementary file 1 — Supporting Information [file ADVS-13-e17378-s001.docx]

**Manipulation of Emergent Collective Excitations via Composition Control in Mixed MPX_3_ Correlated 2D Antiferromagnets**

Cong Tai Trinh^1†^, Na Liu^1†◊^, Rabindra Basnet^2,3^, Dinesh Upreti^2^, Rijan Karkee ^1^, Vigneshwaran Chandrasekaran^1◊^, Andrew C. Jones^1^, Michael T. Pettes^1^, Thuc T. Mai^4,5^, Michael A. Susner^4^, Jin Hu^2,6*^, Rahul Rao^4*^, Han Htoon^1*^

^1^ Center for Integrated Nanotechnologies, Materials Physics and Applications Division, Los Alamos National Laboratory, Los Alamos, NM 87545, USA

^2^ Department of Physics, University of Arkansas, Fayetteville, Arkansas 72701, USA

^3^ Department of Physics, Morgan State University, Baltimore, Maryland 21251, USA
^4^ Materials and Manufacturing Directorate, Air Force Research Laboratory, Wright-Patterson Air Force Base, Ohio 45433, USA

^5^ BlueHalo Inc., Dayton, Ohio 45432, USA

^6^ Institute for Nanoscience and Engineering, MonArk NSF Quantum Foundry, and Smart Ferroic Materials Center, University of Arkansas, Fayetteville, Arkansas 72701, USA

* E-mail: [jinhu@uark.edu; rahul.rao.2@us.af.mil](mailto:jinhu@uark.edu;%20rahul.rao.2@us.af.mil); [htoon@lanl.gov](mailto:htoon@lanl.gov)

† These authors contributed equally.

Supporting Information


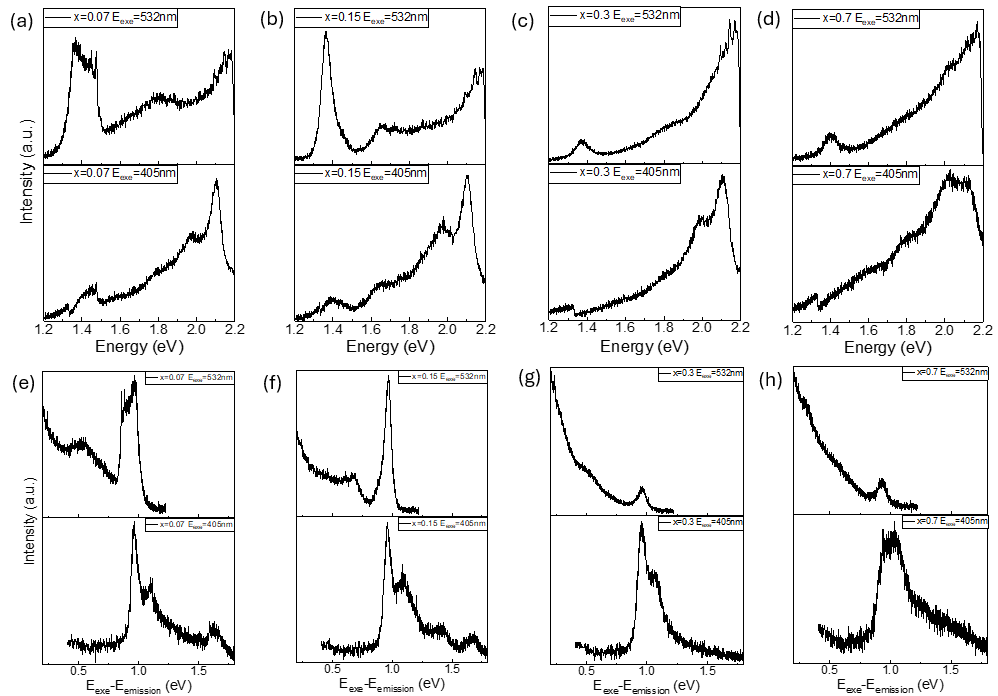


**Figure S1.** PL spectra measured under two excitation wavelengths (532nm and 405nm) for Ni_1-x_Fe_x_PS_3_ with x=0.07(a), 0.15(b), 0.3(c) and 0.7(d). Corresponding wavelength dependent Raman spectra (e-h), extracted from PL energy shift, reveal an electronic Raman scattering peak at 1.0eV energy shift.


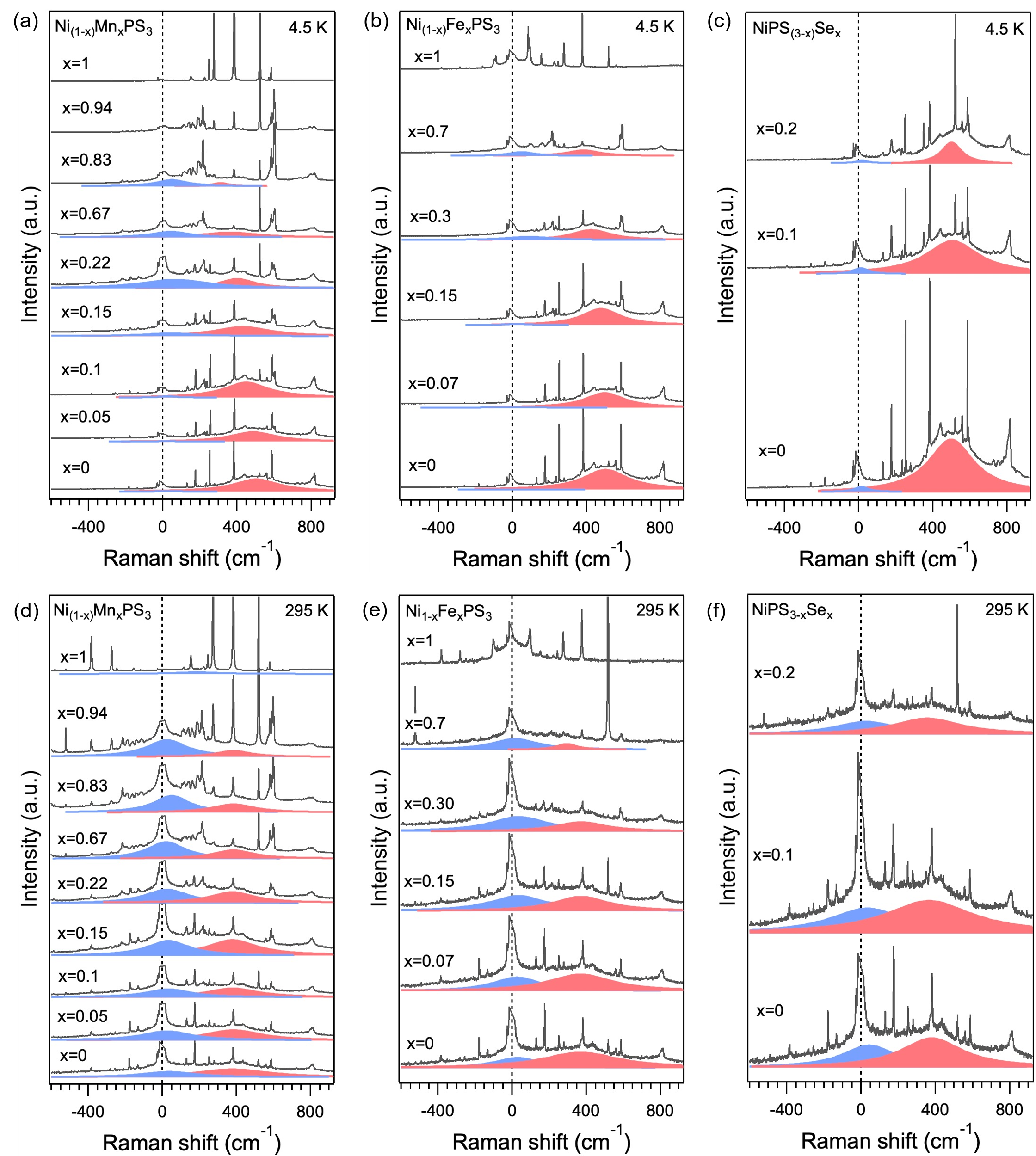


**Figure S2.** (a) – (c) Low T (4.5 K) Raman spectrum of Ni_1-x_Mn_x_PS_3_, Ni_1-x_Fe_x_PS_3_, NiPS_3-x_Se_x_, respectively, with fitted CTC (blue) and 2M (red) continuums. (d) – (f) Corresponding data collected at 295 K.


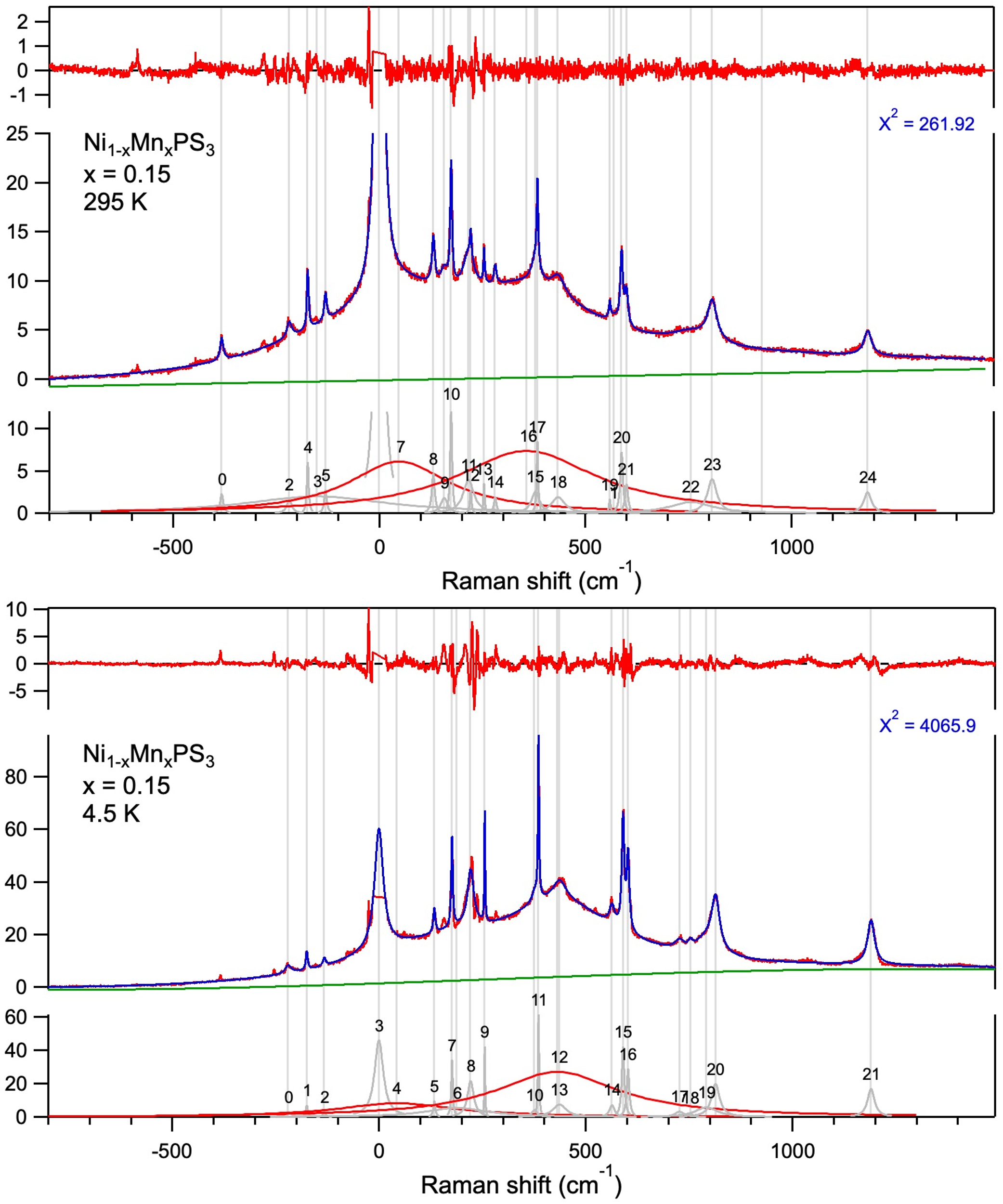


**Figure S3.** Examples of curve fitting (performed using Igor Pro) for spectra collected from Ni_1-x_Mn_x_PS_3_, x=0.15 at 295 K (top) and 4.5 K (bottom). The top traces show the residuals and the individual fitted peaks are shown below the raw spectra. The CTC and 2M peaks are highlighted by their red colors and the rest of the fitted peaks are grey.


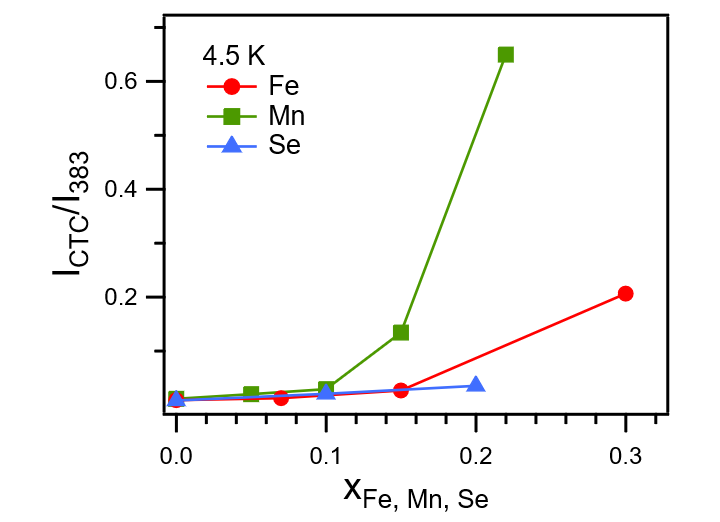


**Figure S4**. Intensity of the CTC peak relative to the 383 cm^-1^ peak.


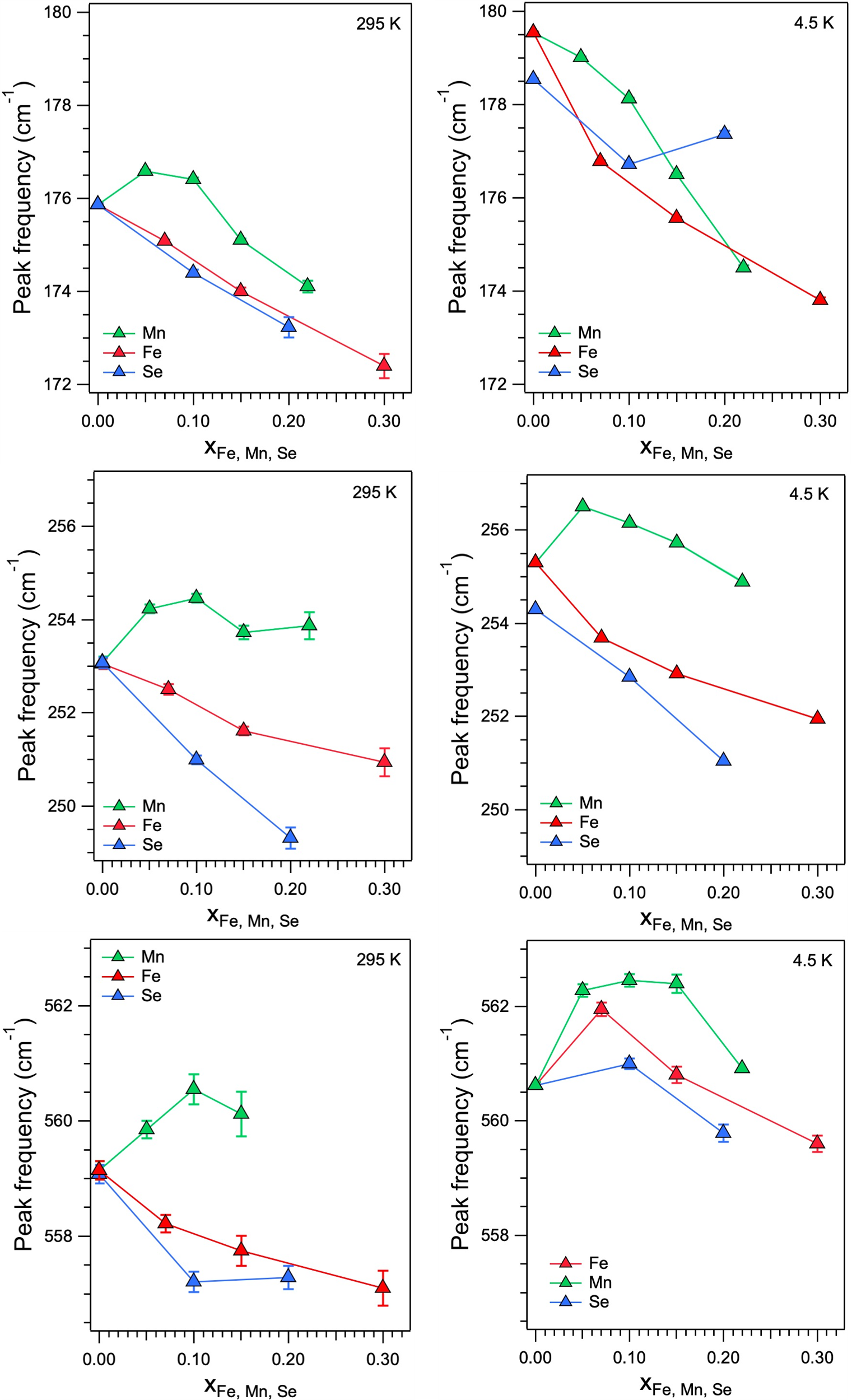


**Figure S5**. Frequencies of the Raman peaks as a function of Mn, Fe and Se concentration at 295 and 4.5 K. In case of Mn substitution, at low concentrations the peaks blueshift in frequency, concomitant with compressive strain in the lattice. However, overall, all peaks redshift in frequency with increasing substituent concentrations, indicating an overall tensile strain imposed on the lattice due to substitution.


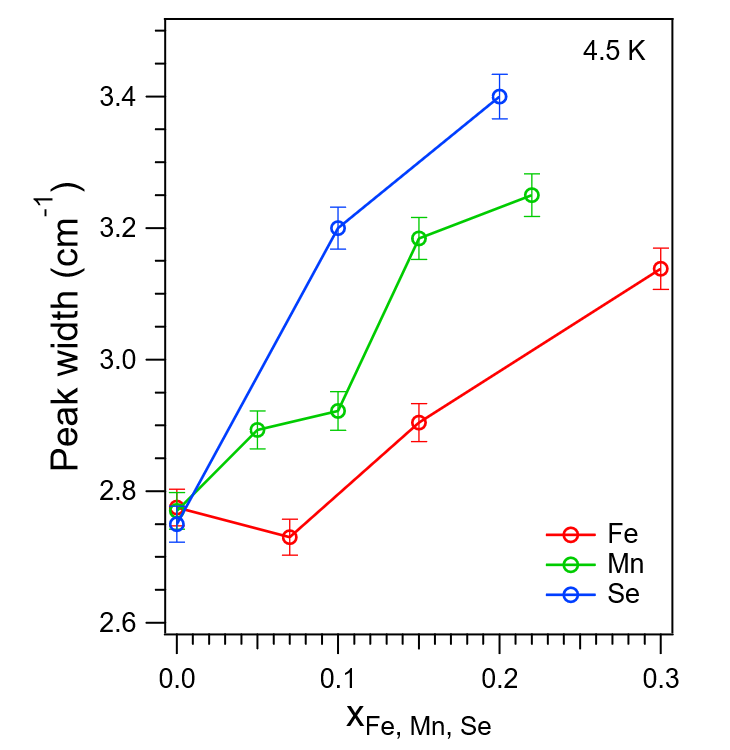


Figure S6. Widths of the 380 cm^-1^ Raman peak as a function of Mn, Fe and Se concentration at 4.5 K. For all substitutions the peak width increases and can be attributed to increasing lattice disorder.


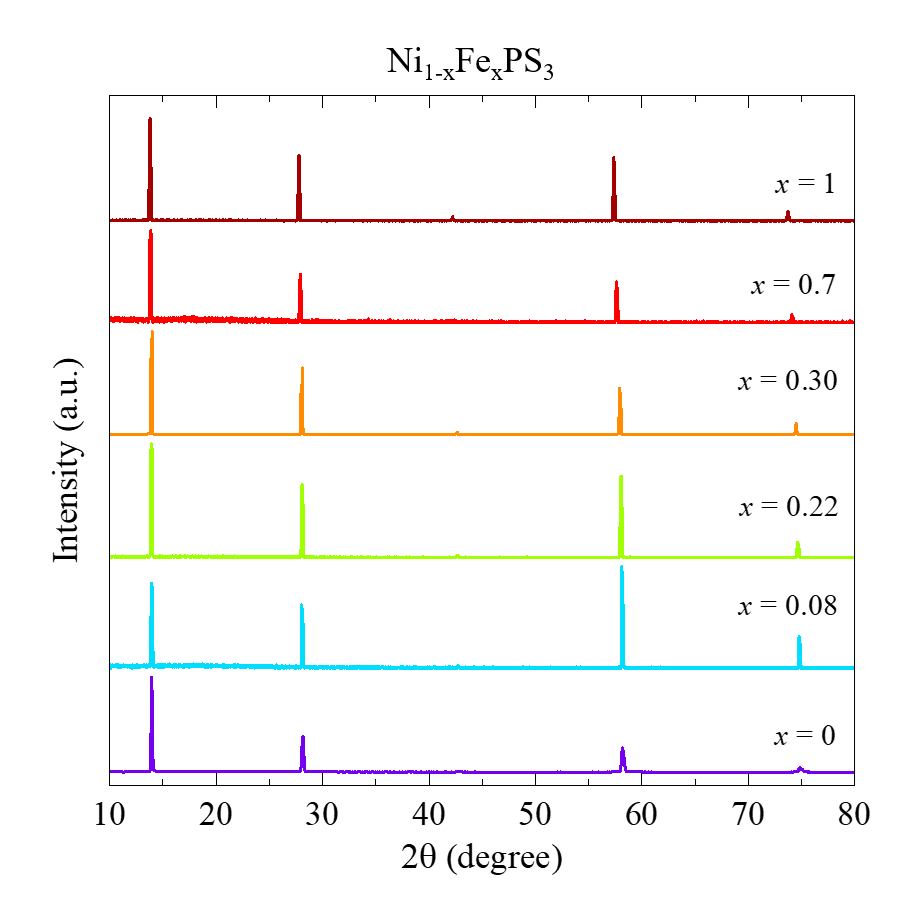


Figure S7. Single-crystal x-ray-diffraction pattern of Ni_1-x_Fe_x_PS_3_ showing the (00L) reflections.

**Table S1** Elementary compositions determined by energy-dispersive X-ray spectroscopy (EDS)

|  | **Composition in main text** | **EDS-determined composition** |
| --- | --- | --- |
| **Ni_1-x_Mn_x_PS_3_** | x = 1 (MnPS_3_) | Mn_1.00_P_0.99±0.07_S_2.98±0.03_ |
|  | x = 0.94 | Ni_0.06±0.005_Mn_0.94±0.04_P_0.83±0.007_S_2.80±0.04_ |
|  | x = 0.83 | Ni_0.17±0.01_Mn_0.83±0.048_P_0.94±0.006_S_2.89±0.006_ |
|  | x = 0.67 | Ni_0.33±0.005_Mn_0.67±0.005_P_0.96±0.012_S_2.93±0.01_ |
|  | x = 0.22 | Ni_0.78±0.08_Mn_0.22±0.008_P_0.95±0.0122_S_2.84±0.06_ |
|  | x = 0.15 | Ni_0.85±0.04_Mn_0.15±0.03_P_0.85±0.05_S_2.87±0.09_ |
|  | x = 0.10 | Ni_0.90±0.03_Mn_0.10±0.03_P_0.98±0.03_S_2.96±0.08_ |
|  | x = 0.05 | Ni_0.95±0.04_Mn_0.05±0.0009_P_0.97±0.03_S_2.84±0.02_ |
|  | x = 0 (NiPS_3_) | Ni_1.00_P_1.01±0.04_S_3.06±0.04_ |
| **Ni_1-x_Fe_x_PS_3_** | x = 1 (FePS_3_) | FeP_1.02±0.01_S_2.98±0.01_ |
|  | x = 0.70 | Ni_0.30±0.015_Fe_0.70±0.006_P_0.92±0.005_S_2.94±0.02_ |
|  | x = 0.30 | Ni_0.70±0.04_Fe_0.30±0.008_P_0.89±0.01_S_2.92±0.02_ |
|  | x = 0.15 | Ni_0.85±0.024_Fe_0.15±0.001_P_1.12±0.005_S_3.17±0.03_ |
|  | x = 0.07 | Ni_0.93±0.012_Fe_0.07±0.007_P_1.07±0.06_S_3.07±0.06_ |
| **Ni_1-x_Cr_x_PS_3_** | x = 0.09 | Ni_0.91±0.017_Cr_0.09±0.0007_P_0.93±0.04_S_2.98±0.003_ |
|  | x = 0.03 | Ni_0.97±0.056_Cr_0.03±0.004_P_0.89±0.02_S_2.93±0.04_ |
|  | x = 0.016 | Ni_0.984±0.047_Cr_0.016±0.001_P_0.95±0.012_S_2.83±0.04_ |
| **NiP(S_3-x_Se_x_)** | x = 0.20 | Ni_1±0.08_P_0.92±0.03_S_2.80±0.08_Se_0.20±0.04_ |
|  | x = 0.10 | Ni_1±0.07_P_0.99±0.03_P_2.90±0.06_S_0.10±0.02_ |
